# The impact of gut microbiota on cervical cancer and precancerous lesions: neglected status, mechanisms, challenges, and a call to action

**DOI:** 10.3389/fimmu.2026.1826283

**Published:** 2026-05-28

**Authors:** Yanfang Li, Jingrui Zhang, Songxiao Wu, Zengbin Liu, Shan Qin

**Affiliations:** 1 Department of Gynecology, The Second Hospital of Hebei Medical University, Shijiazhuang, Hebei, China; 2 Department of Clinical Laboratory, The Fourth Hospital of Shijiazhuang, Shijiazhuang, Hebei, China; 3 Department of Clinical Laboratory, The Second Hospital of Hebei Medical University, Shijiazhuang, Hebei, China

**Keywords:** cervical cancer, fecal microbiota transplantation, gut microbiota, HPV clearance, immune regulation, short-chain fatty acids, vaginal microecology

## Abstract

Cervical cancer (CC) remains a major global health threat closely associated with persistent high-risk human papillomavirus (HPV) infection. Although immune checkpoint inhibitors (ICIs) have emerged as a therapeutic option, their objective response rates remain unsatisfactory. Variations in the local vaginal microbiota alone cannot fully explain inter-individual differences in HPV clearance, suggesting that additional systemic immune determinants are involved. The gut microbiota, a central regulator of host systemic immunity, can profoundly influence HPV clearance and antitumor immune responses by shaping dendritic cell (DC) function, modulating the Th1/Th2 balance, regulating regulatory T cell (Treg) expansion, and affecting natural killer (NK) cell activity. Emerging evidence indicates that specific gut microbial taxa are causally associated with HPV infection, cervical intraepithelial neoplasia (CIN), and cervical cancer, and may reshape the vaginal microecological environment and enhance immunotherapy responses. However, this dimension has long received insufficient attention. This Perspective systematically addresses four core issues: the neglected status of gut microbiota research and the functional boundaries of vaginal microecology; key mechanisms through which gut microbiota regulate HPV clearance and cervical lesion progression; major challenges restricting progress; and potential strategies for promoting clinical translation. This work aims to establish a theoretical framework for gut microbiota–based interventions in cervical cancer prevention and treatment, providing directional guidance for this emerging interdisciplinary field.

## 1 Introduction

Cervical cancer is the fourth most common malignancy among women worldwide, with approximately 662,000 new cases and 349,000 deaths reported annually (1). The disease is primarily associated with persistent infection with high-risk human papillomavirus (HPV). Although approximately 80–90% of HPV infections are spontaneously cleared by the host immune system within 24 months after infection, 10–20% of individuals fail to eliminate the virus, resulting in persistent infection that may lead to cervical intraepithelial neoplasia (CIN) and progressive malignant transformation, ultimately culminating in cervical cancer (2). These observations highlight the critical importance of effective HPV clearance following infection.

From a therapeutic perspective, immune checkpoint inhibitors (ICIs) have gradually become an important component of cervical cancer treatment. However, their clinical efficacy remains limited. Studies have shown that the objective response rate of pembrolizumab in patients with previously treated recurrent or metastatic cervical cancer is only 12.2–14.3% (3). This limited response may partly be associated with dysbiosis of the vaginal microbiota following HPV infection (4, 5). Nevertheless, even among individuals with highly similar vaginal microbial profiles, significant differences remain in HPV clearance capacity and disease outcomes (6, 7). This paradox suggests that focusing solely on the local vaginal microenvironment is insufficient to explain inter-individual variability in HPV clearance and underscores the need to reconsider the problem from the perspective of systemic immunity.

Accumulating evidence indicates that the host systemic immune status plays a decisive role in determining whether HPV can be effectively eliminated. Viral clearance following HPV infection largely depends on host systemic immune responses, particularly dendritic cell–mediated adaptive T-cell responses and the cytotoxic activity of natural killer (NK) cells, which represent critical steps in viral elimination (8, 9). In addition, peripheral immune parameters—including the balance between Th1 and Th2 responses, the expansion of regulatory T cells, and the exhaustion status of NK cells—directly influence HPV clearance efficiency and responses to immune checkpoint therapy (10, 11). These findings highlight the pivotal role of systemic immune competence in both HPV elimination and immunotherapy outcomes. Importantly, systemic immune status is strongly influenced by the gut microbiota, which serves as a key regulator of host immune homeostasis.

Recent studies further demonstrate that specific gut microbial taxa are causally associated with HPV infection, CIN, and cervical cancer, with certain microbial communities exhibiting protective effects (12, 13). In addition, the gut microbiota may contribute to reshaping the vaginal microecological environment, promoting clearance of high-risk HPV (HR-HPV), and enhancing immune responses to HPV-related therapies (14, 15). Despite these emerging insights, the regulatory role of the gut microbiota in this context has often been overlooked, and its clinical translation still faces numerous challenges.

To better elucidate the role of the gut microbiota in shaping the cervicovaginal immune microenvironment, this Perspective organizes its analysis around four interrelated compartments: the *vaginal vault microbiota*, *intralesional immunity*, *systemic immunity*, and the *gut microbiota*, which communicate through a “gut–systemic–mucosal/intralesional” axis. Importantly, peripheral circulating immune cells do not necessarily achieve effective infiltration into cervical lesions; whether they can traverse stromal barriers, resist local exhaustion signals, and exert cytotoxic function depends on chemokine gradients and the local density of immunosuppressive cells (16, 17). The gut microbiota primarily sets the systemic immune baseline through SCFAs and the cGAS–STING–IFN-I axis, thereby indirectly influencing the other three compartments.

**Figure 1:**
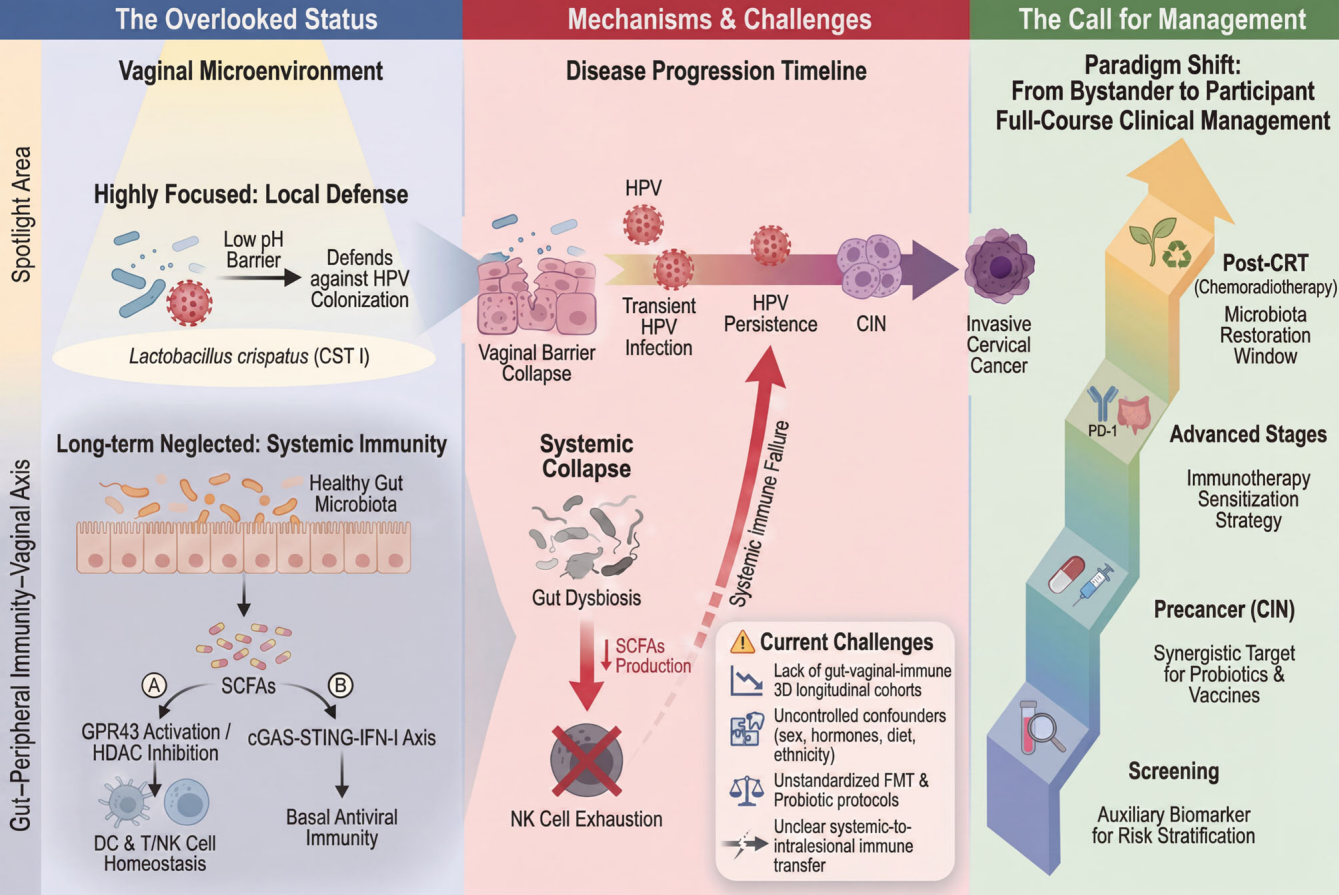
The overlooked role of gut microbiota in cervical cancer: status, mechanisms, challenges, and call for action. This graphical abstract adopts a horizontal three-panel progressive layout to illustrate the core conceptual framework of this study. The left panel (The Overlooked Status) highlights the current imbalance in research focus. The upper “spotlight” area represents the well-studied local vaginal microenvironment, where *Lactobacillus crispatus*–dominated CST I maintains a low-pH barrier that protects against initial HPV colonization. In contrast, the lower “shadow” area depicts the largely overlooked gut microbiota. A healthy gut microbiome secretes short-chain fatty acids (SCFAs) that regulate systemic immunity through two principal pathways: **(A)** activation of GPR43 and inhibition of HDAC, which maintain dendritic cell and T/NK cell homeostasis; and **(B)** activation of the cGAS–STING–IFN-I axis, establishing basal antiviral immunity. Together, these pathways form the immunological foundation of the “gut–peripheral immune–vaginal/intralesional” axis.

To systematically advance both the theoretical understanding and clinical translation of gut microbiota in the prevention and treatment of HPV infection and cervical cancer, this Perspective focuses on four key scientific questions (**Figure 1**):

1. What is the current status of gut microbiota research in HPV infection and cervical disease, and where are the functional boundaries between gut microbiota and vaginal microecology?
2. Through what mechanisms does the gut microbiota influence HPV clearance and the progression of cervical lesions?
3. What are the major challenges currently facing this field, and how do they restrict its development? How might these barriers be overcome?
4. How can gut microbiota research move beyond its current limitations, what specific strategies are required, and what potential impacts may arise from these advances?

## 2 Current neglect of the microbiota in cervical carcinogenesis

The female vaginal microbiota can be classified into five community state types (Community State Types, CST): CST I (dominated by *Lactobacillus crispatus*), CST II (*L. gasseri*), CST III (*L. iners*), CST V (*L. jensenii*), and CST IV (high diversity with low abundance of *Lactobacillus*) (5). In healthy individuals, the vaginal vault microbiota is typically dominated by *Lactobacillus*, which maintains a low vaginal pH (<4.5) through lactic acid production, thereby preventing pathogen colonization (18). Studies have demonstrated that a high abundance of *L. crispatus* is significantly associated with a reduced risk of cervical intraepithelial neoplasia (CIN) (OR = 0.50, 95% CI 0.29–0.88), highlighting the critical protective role of the lactobacillus barrier in suppressing CIN progression (19).

When the dominance of *Lactobacillus* is disrupted, the microbiota often shifts from CST I toward CST III or CST IV, leading to impairment of the lactic acid barrier and the development of microbial dysbiosis. This shift results in reduced mucosal resistance, a 3–5-fold increase in HPV infection risk, and a 2–3-fold increase in the risk of high-grade lesions and cervical cancer (5). These findings suggest that the disease trajectory from health to HPV infection, CIN, and eventually cervical cancer closely parallels the ecological transition of vaginal microbiota from CST I/II → CST III → CST IV (5, 20). This transition exerts cumulative effects across three dimensions: barrier disruption, pathogen expansion, and inflammatory activation. Dysbiosis leads to breakdown of the lactate barrier and abnormal elevation of vaginal pH (>4.5), which may partly explain why abnormal vaginal pH is observed in nearly 100% of invasive cervical cancer (ICC) cases (21). Barrier dysfunction also facilitates colonization by opportunistic pathogens such as Mycoplasmatales (22), further aggravating microbial imbalance and promoting increased levels of pro-inflammatory cytokines (IL-6, IL-8, TNF) as well as immunosuppressive mediators such as IL-10 at the local vaginal mucosa. These immune alterations prolong the clearance time of high-risk HPV and increase the probability of persistent infection (23, 24).

Meanwhile, HPV16/18 E6/E7 oncoproteins can directly upregulate IL-6 expression in keratinocytes (25), thereby inducing Th17 differentiation and promoting IL-17 production. This process generates a positive feedback loop that amplifies inflammatory signaling while simultaneously suppressing host anti-HPV immune responses, ultimately exacerbating viral persistence (26). Thus, a typical circular mechanism emerges in which barrier disruption and pathogen expansion activate inflammatory pathways, while inflammation further impairs viral clearance. This cycle can transform transient infections into persistent infections (23, 26, 27), increasing the risk of high-grade lesions or cervical cancer by approximately 1.5–2-fold and complicating therapeutic management (4, 28, 29).

Even among individuals with broadly similar microbial compositions, significant heterogeneity exists in HPV clearance and disease outcomes. Studies have shown that the vaginal microbiota of CIN patients often undergoes a process of “re-lactobacillization” after surgical treatment, characterized by reduced abundance of *Atopobium vaginae* and decreased levels of pro-inflammatory cytokines such as TNF-α and IL-1β, which may facilitate viral clearance (6, 30). However, some patients fail to achieve stable microbial reconstruction, suggesting the presence of additional modulatory factors. Elevated inflammatory mediators such as IL-1β and IP-10 may partially explain these differences (31). Moreover, although *L. iners* abundance is associated with HPV clearance in some HPV-positive populations, it is paradoxically linked to persistent HPV infection in CIN patients receiving non-surgical treatment (OR = 3.94, p = 0.041) (32). This apparent contradiction indicates that the predictive value of *L. iners* may be context-dependent.

Host immune genetics further contribute to this heterogeneity. HLA class I and II polymorphisms can alter the binding and presentation of viral peptides such as E6/E7, thereby modulating the magnitude of HPV-specific T-cell responses (33). Consequently, complex interactions between HLA alleles and HPV genotypes may occur, whereby the same allele may facilitate clearance of certain HPV subtypes while impairing clearance of others (34–36), indicating that host systemic immunity also contributes to HPV clearance. In addition, host genetic background can influence the baseline composition of the vaginal microbiota. For example, the cervical microbiota of Puerto Rican women is frequently dominated by *L. iners*, whereas Native American women exhibit a higher prevalence of non-*Lactobacillus*-dominated communities, both independent of HPV infection status (37, 38). Collectively, these findings indicate that HPV infection and clearance are shaped by a multidimensional interaction among the vaginal vault microbiota, host genetics, and systemic immunity.

Beyond the vaginal microbiota, the gut microbiota also plays an important but often overlooked role in HPV clearance. The *Lactobacillus*-dominated vaginal barrier primarily functions to prevent HPV acquisition (39), whereas viral clearance after infection largely depends on systemic immunity, which is strongly regulated by the gut microbiota (40). A healthy gut microbiota produces sufficient short-chain fatty acids (SCFAs) that enter the circulation via the portal vein and activate immune-regulatory pathways including GPR43/GPR109A signaling, histone deacetylase (HDAC) inhibition, and mTOR–S6K metabolic reprogramming. These pathways help maintain the functional balance of dendritic cells and macrophages and regulate the differentiation of regulatory T cells (Treg) and effector T cells, thereby preserving systemic immune homeostasis (41–43).

However, HPV infection is often accompanied by gut microbiota dysbiosis, leading to reduced SCFA production and disruption of immune regulatory pathways. As a result, systemic immune equilibrium is disturbed and antiviral immune responses are weakened, ultimately impairing HPV clearance. Concurrently, vaginal microbial imbalance such as bacterial vaginosis (BV) can produce similar SCFA metabolites, which may further elevate vaginal pH and exacerbate BV-associated dysbiosis (44–46). These processes collectively suppress immune responses and hinder viral clearance. In addition, reductions in gut microbial α-diversity following infection have been associated with increased exhaustion of peripheral NK cells, further contributing to systemic immunosuppression (9). This mechanism may partly explain why mice exhibiting robust responses to HPV16 E7 vaccination show significantly higher abundance of *Bacteroides* in the gut, whereas non-responders are enriched in Proteobacteria (47).

Furthermore, a healthy gut microbiota can release extracellular vesicle DNA that activates the cGAS–STING–type I interferon axis, thereby establishing a systemic antiviral “basal tone” (48). The microbiota can also activate conventional dendritic cells (cDCs) and promote their migration to tumor sites, facilitating CD8^+^ T-cell responses and enhancing antiviral immunity (49). In contrast, high-risk HPV infection not only disrupts this immune axis and reduces type I interferon signaling but also produces E6/E7 oncoproteins that alter intralesional epidermal dendritic cell recruitment and localization (8, 50). These combined effects weaken host immune surveillance and favor viral persistence.

Taken together, these findings highlight the crucial role of the gut microbiota in HPV clearance. Nevertheless, direct empirical validation of the complete “gut microbiota–peripheral immunity–vaginal microbiota” axis remains limited. Only preliminary evidence suggests that gut microbial status may partially buffer local inflammation in individuals with vaginal dysbiosis (51, 52).

**Table 1** presents a four-compartment comparison across the *vaginal vault microbiota*, *intralesional immunity*, *systemic immunity*, and the *gut microbiota*. **Table 2** summarizes the core synergistic effects of the local microenvironment and systemic immunity at different disease stages. **Table 3** presents systemic immune compartment (peripheral blood) indicators, their consistency with intralesional immunity, and their clinical significance.

**Table 1:** Four-compartment comparison: vaginal vault microbiota, intralesional immunity, systemic immunity, and gut microbiota.

Dimension	Vaginal vault microbiota	Intralesional immunity	Systemic immunity	Gut microbiota	Ref.
Core role	Mucosal barrier; defense against initial HPV colonization	TIL-mediated local clearance; suppressed by E6/E7	Th1/NK effector “chassis”	Sets the systemic immune threshold (via SCFAs)	(9, 18, 21, 42, 43, 47)
Protective marker	CST I; pH < 4.5	High TIL density; low PD-L1	High Th1; low Treg; low NLR	High α-diversity; *Bacteroides* enrichment	(5, 9, 18, 19, 47, 82)
Risk marker	CST IV; pH > 4.5; *Gardnerella* enrichment	Low TIL; high PD-L1; increased intratumoral microbial heterogeneity	Treg expansion; NK exhaustion (TIGIT/KLRG1)	Low diversity; Proteobacteria enrichment	(4, 18, 47, 83–85)
Impact on HPV/lesion	Defends initial colonization	Determines efficacy of local clearance	Determines circulating cytotoxic capacity	Indirectly regulates the above three	(6, 24, 44, 51)
Key limitation	Poorly reflects systemic state	Difficult to sample (biopsy required)	≠ actual intralesional immunity	Downstream transfer efficiency unclear	(16, 17)

This table provides a side-by-side comparison of the four interrelated compartments that together govern HPV clearance and cervical lesion progression, covering their core roles, protective and risk-associated markers, impact on disease, and key limitations. The “Key limitation” row emphasizes that no single compartment adequately reflects the others, underscoring the need for an integrated four-compartment framework rather than reliance on any one dimension alone.

CST, community state type; HPV, human papillomavirus; TIL, tumor-infiltrating lymphocyte; PD-L1, programmed death-ligand 1; Th1, type 1 helper T cell; Treg, regulatory T cell; NLR, neutrophil-to-lymphocyte ratio; TIGIT, T-cell immunoreceptor with Ig and ITIM domains; KLRG1, killer cell lectin-like receptor G1; SCFAs, short-chain fatty acids. Symbol: “≠” denotes “not equivalent to”.

**Table 2:** Core synergistic effects of the local microenvironment and systemic immunity at different stages.

Disease stage	Core four-compartment synergistic effect (one-line summary)	Ref.
Initial infection	An intact vaginal vault barrier combined with systemic immunity (DC maturation and gut-derived SCFA-induced Treg/anti-inflammatory baseline) jointly determines whether the virus is recognized and cleared early	(39, 40, 42, 48)
Persistent infection	Gut dysbiosis induces systemic immune exhaustion, compounded by abnormal vaginal SCFAs driving local inflammation; both prevent viral clearance	(9, 18, 44–46)
Precancerous lesions (CIN)	Local metabolic disruption and systemic low-grade chronic inflammation synergistically accelerate epithelial malignant transformation	(40, 86, 87)
Invasive cervical cancer (ICC)	Collapse of systemic immune surveillance (NK exhaustion, sharp loss of gut α-diversity) + intralesional DC function suppressed by E6/E7 + increased intratumoral microbial heterogeneity together drive tumor immune evasion	(8, 9, 88)

This table distills, for each stage of the HPV–CIN–cervical cancer continuum, the dominant synergistic interaction among the four compartments into a single integrative statement. It illustrates how disease progression is driven not by the failure of any single compartment but by the cumulative uncoupling of vaginal barrier, intralesional immunity, systemic immunity, and gut microbiota.

DC, dendritic cell; NK, natural killer cell; CIN, cervical intraepithelial neoplasia; ICC, invasive cervical cancer; E6/E7, HPV early oncoproteins 6 and 7; SCFA, short-chain fatty acid; Treg, regulatory T cell; Th1/Th2, type 1/type 2 helper T cells; NLR, neutrophil-to-lymphocyte ratio; PLR, platelet-to-lymphocyte ratio.

**Table 3:** Systemic immune compartment (peripheral blood) indicators and their relationship with intralesional immunity.

Immune indicator	HPV clearance	HPV infection/CIN progression	Consistency with intralesional immunity	Clinical significance	Ref.
Peripheral T/NK ratio	T↑; NK↓	T↓; NK↑ but functionally exhausted	Moderate (peripheral NK expansion may not reflect intratumoral function)	NK overexpansion with impaired function suggests adaptive immunity–dominated clearance	(11, 89)
Th1 response (IFN-γ, IL-2)	Significantly upregulated after HPV stimulation	No significant Th1 enhancement	Relatively high	A functional Th1 response is a core mechanism of viral clearance	(90)
Treg (CD4^+^CD25^hi^)	No significant increase	Significantly elevated in peripheral blood	High (systemic and intratumoral Treg often co-elevate)	Treg expansion promotes viral persistence and immunosuppression	(10)
NLR/PLR/SIRI	Low levels	High levels (PLR > 118.4; SIRI ≥ 0.57)	Indirect (reflects systemic inflammatory burden)	Predicts CIN2+ recurrence risk	(91, 92)
NK inhibitory receptors (TIGIT, KLRG1)	Low expression	CD56^bright^ NK high expression; IFN-γ ↓	High (peripheral exhaustion often accompanies intratumoral exhaustion)	NK exhaustion is an early signal of persistent infection and progression	(11)

This table summarizes peripheral blood immune indicators associated with HPV clearance or disease progression and, crucially, specifies the degree to which each peripheral readout reliably mirrors intralesional immune status. It reminds the reader that the translational value of systemic biomarkers varies by parameter, supporting the argument that peripheral measurements should be interpreted in conjunction with, rather than as a substitute for, lesion-level assessment.

T, T lymphocyte; NK, natural killer cell; IFN-γ, interferon-gamma; IL-2, interleukin-2; CD4^+^CD25^hi^, CD4-positive CD25-high regulatory T cells; SIRI, systemic inflammation response index; CIN2+, cervical intraepithelial neoplasia grade 2 or worse; CD56^bright^, CD56-high-expressing NK cell subset; HPV, human papillomavirus. Symbols: ↑, increased; ↓, decreased; the superscripts “hi” and “bright” denote high surface expression.

## 3 Core challenges

### 3.1 Insufficient longitudinal validation cohorts

Although several two-sample Mendelian randomization (MR) studies have supported causal associations between specific microbial taxa and cervical cancer risk at the level of genetic instrumental variables—for example, *Actinomyces* appears protective (OR ≈ 0.52–0.55), whereas Lachnospiraceae UCG001 is associated with increased risk (OR ≈ 2.0) (53)—stratified MR analyzes have also identified 17 microbial taxa with stage-specific effects across HPV infection, cervical intraepithelial neoplasia (CIN), and cervical cancer (13). In addition, bidirectional causal relationships have been reported, suggesting that cervical cancer itself may alter gut microbiota composition (12, 54). Longitudinal studies have further documented dynamic shifts in the vaginal microbiome during the progression from HPV infection to CIN (7, 55, 56). However, no prospective cohort to date has simultaneously collected gut–vaginal–peripheral immune multi-dimensional data throughout the full disease trajectory from HPV infection to CIN and ultimately cervical cancer (15). Consequently, a critical gap remains in longitudinal validation cohorts, which urgently needs to be addressed.

### 3.2 Difficulty in controlling confounding factors

Multiple confounding variables influence both local and intestinal microbiota, and many of these factors are difficult to control. Studies have shown that sexual behavior, smoking, alcohol consumption, and psychological stress are significantly associated with increased vaginal microbial diversity and enrichment of bacterial vaginosis (BV)–associated taxa, whereas hormonal contraception and appropriate personal hygiene practices tend to maintain vaginal microbial homeostasis (57). The menstrual cycle and its different phases also represent major confounders in vaginal microbiome studies; notably, serum estradiol levels are significantly positively correlated with *Lactobacillus crispatus* abundance (r = 0.11, p < 0.001) (58). Dietary factors may further contribute, as sugar intake has been linked to variations in *L. crispatus* abundance and CST IV–associated taxa (59). In addition, antibiotic exposure can simultaneously disrupt both gut and vaginal microbiota and potentially alter the trajectory of cervical tumorigenesis (60). Other confounders, such as racial or ethnic background—an independent prognostic factor—may also influence microbial composition (61). The interaction and accumulation of these variables often lead to inconsistent findings across studies, such as the apparent “protective–risk” dual role reported for *L. iners*. Therefore, future research should adopt multivariable modeling strategies and high-quality randomized controlled designs to better control major confounders and clarify underlying mechanisms.

### 3.3 Lack of standardization in gut microbiota interventions

Currently, consensus is lacking regarding donor screening criteria, stool preparation protocols, routes of administration (e.g., enema, capsules, or nasojejunal tube), and treatment regimens for fecal microbiota transplantation (FMT). Similarly, probiotic interventions remain poorly standardized with respect to strain selection, dosage, and treatment duration (62). As a result, substantial heterogeneity exists across studies. While FMT demonstrates excellent efficacy in acute intestinal infections such as recurrent *Clostridioides difficile* infection, with response rates exceeding 90%, its effectiveness in extra-intestinal diseases is markedly lower and often difficult to reproduce (63). Moreover, the composition of the gut microbiota varies substantially between individuals and is shaped by multiple factors including diet, geography, host genetics, and immune status (64). These variations impose fundamental limitations on the generalizability of microbiota-based interventions, indicating that considerable progress is still required before precise and individualized microbiome-targeted therapies can be widely implemented.

### 3.4 Uncertain transfer efficiency between systemic and intralesional immunity

Although the gut microbiota primarily modulates the systemic immune baseline, the relationship between peripheral immune indicators and intralesional immune status nonetheless warrants careful consideration. In HPV16-related CIN, peripheral CD56^bright^ NK cells have been shown to display an exhausted phenotype with high TIGIT/KLRG1 expression (11), while intratumoral E6/E7 oncoproteins can further suppress DC recruitment and intensify stromal immune exclusion, thereby hindering peripheral effector cell infiltration (8, 50). Such decoupling between peripheral and intralesional immunity has been extensively documented in tumor immunology (16, 17). This underscores the need for future studies to simultaneously collect, where feasible, peripheral blood, vaginal secretions, and lesion tissue samples, so as to validate the actual efficiency with which gut microbiota–driven regulation is transmitted to the lesion site.

## 4 Discussion

Over the next 5–10 years, distinct strategies will be required to address the four core challenges outlined above. To overcome the lack of longitudinal validation cohorts, large-scale prospective cohorts spanning the entire trajectory from initial HPV infection to malignant transformation should be established from a causal inference perspective. These cohorts should include synchronized sampling of gut microbiota, vaginal vault microbiota, and peripheral systemic immune indicators (with the addition of lesion tissue biopsies where feasible), to elucidate the dynamic causal relationships between microbial succession and disease progression and to validate the transfer efficiency between systemic and intralesional immunity (15). Methodologically, integrative frameworks combining two-sample or bidirectional MR analyzes with colocalization analysis, time-dependent statistical models, and deep learning approaches may provide multi-level causal validation (65, 66). In addition, global multicenter cohorts across diverse ethnic populations are necessary to identify universally relevant pathogenic and protective microbial taxa (38, 67, 68). Experimental approaches using germ-free animal models and organoid systems may further clarify the molecular pathways through which gut-derived metabolites cross anatomical barriers and influence cervical immune cells (40, 69), thereby providing a theoretical basis for the future standardization of microbiome-based interventions.

Where ethically permissible, randomized controlled trials (RCTs) should also be conducted to rigorously control major confounders and systematically evaluate the benefit–risk profile of probiotics and FMT in individuals with cervical precancerous lesions (70). Such trials would help identify the most relevant probiotic strains and guide the rational selection of microbial formulations.

Emerging evidence suggests that gut microbial α-diversity combined with natural killer (NK) cell exhaustion scores may assist in identifying individuals with a high risk of CIN progression (9). Moreover, multiple RCTs have demonstrated that oral administration of *L. crispatus* M247 can increase HR-HPV clearance rates to approximately 60% (71). In parallel, the oral lactobacillus-based vaccine IGMKK16E7 has shown promising efficacy in a phase II RCT, significantly promoting regression of CIN2/3 lesions (72). These findings suggest that integrating gut microbiota indicators with vaginal pH levels and local immune markers could enable the development of a “local–systemic–metabolic” multidimensional risk stratification model to predict disease progression and guide clinical management (9, 67).

In addition, accumulating evidence indicates that gut microbiota composition is a key determinant of immune checkpoint inhibitor (ICI) efficacy. Through FMT, beneficial microbial communities from responders can be transferred to non-responders, potentially reversing resistance to immunotherapy (73, 74). In cervical cancer specifically, gut dysbiosis has been associated with peripheral (systemic immune compartment) NK cell exhaustion and poorer clinical outcomes (9). Persistent microbial imbalance may further impair antitumor lymphocyte activity, thereby reducing sensitivity to subsequent immunotherapy (75, 76). These findings highlight the potential value of routine gut microbiota assessment prior to immunotherapy, as well as the implementation of structured microbiota restoration strategies following radiotherapy or chemotherapy to mitigate immune resistance (77).

We therefore strongly advocate incorporating gut microbiota assessment and intervention into the comprehensive clinical management of HPV infection and cervical cancer. During the screening stage, microbiota-derived indicators could serve as auxiliary biomarkers for risk stratification. In precancerous stages, probiotics or microbiota-based vaccines may function as synergistic interventions to enhance HPV clearance. In advanced disease, microbiota modulation could serve as an immunotherapy-sensitizing strategy to overcome resistance, while post-treatment microbiota restoration may improve long-term outcomes (**Tables 4**, **5**).

**Table 4:** Clinical application of gut microbiota in cervical cancer prevention and screening.

Application stage	Core intervention strategy	Expected clinical benefit	Ref.
Screening and early warning	Gut α-diversity assessment combined with NK cell exhaustion scores (e.g., TIGIT/KLRG1)	Identification of individuals at high risk of persistent HPV infection and CIN progression, enabling precision stratification and early intervention	(9, 93, 94)
Adjunct to vaccination	Supplementation with specific probiotics (e.g., *L. crispatus* M247, *Bacteroides* spp.) combined with HPV immunization	Increased HPV clearance rates; enhanced T-cell responses induced by therapeutic vaccines	(47, 70–72)
Precision nutritional intervention	Mediterranean diet patterns and PUFA supplementation; modulation of the “estrobolome”	Regulation of chronic inflammation and estrogen homeostasis via microbial metabolites, improving the immune microenvironment	(40, 98–100)

This table outlines how gut microbiota–based strategies may be integrated into the preventive and early-detection phases of cervical cancer management, spanning microbial biomarkers for risk stratification, probiotic adjuncts to vaccination, and precision nutritional modulation. These applications collectively aim to enhance HPV clearance and delay or prevent the transition from persistent infection to precancerous lesions.

PUFA, polyunsaturated fatty acid; HPV, human papillomavirus; CIN, cervical intraepithelial neoplasia. Term: “estrobolome” refers to the collective gut microbial genes encoding enzymes (e.g., β-glucuronidases) that metabolize estrogens.

**Table 5:** Clinical application of gut microbiota in cervical cancer treatment and recovery.

Application stage	Core intervention strategy	Expected clinical benefit	Ref.
Postoperative recovery management	Correction of gut dysbiosis; reduction of systemic inflammatory markers (eotaxin, NLR, PLR)	Promotion of cervical mucosal repair; reduction of HPV persistence and recurrence risk	(30, 40, 91, 92)
Combination immunotherapy in advanced disease	Modulation of gut microbiota (probiotics or FMT) combined with PD-1/CTLA-4 inhibitors (e.g., FMT + cadonilimab trials)	Reversal of immune exhaustion; improved ICI efficacy; overcoming therapeutic resistance	(9, 73, 74, 95, 96)
Microbiota restoration after chemoradiotherapy	Probiotic or FMT intervention to correct treatment-induced gut dysbiosis	Restoration of microbial diversity; improved subsequent immunotherapy responsiveness	(75, 97)

This table summarizes gut microbiota–directed strategies applicable to the therapeutic and post-treatment phases of cervical cancer care, including postoperative microbiome repair, combination immunotherapy sensitization, and microbial restoration after chemoradiotherapy. Together, these interventions target immune exhaustion, checkpoint-inhibitor resistance, and long-term recovery outcomes.

FMT, fecal microbiota transplantation; PD-1, programmed cell death protein 1; CTLA-4, cytotoxic T-lymphocyte-associated protein 4; ICI, immune checkpoint inhibitor; NLR, neutrophil-to-lymphocyte ratio; PLR, platelet-to-lymphocyte ratio; eotaxin, CCL11 chemokine; HPV, human papillomavirus.

For patients undergoing treatment for HPV infection or cervical cancer, risk stratification based on the degree of immunosuppression—such as recommended in the 2024 clinical practice guidelines of the American Gastroenterological Association (AGA)—may further guide microbiota-targeted interventions (78). Individuals with normal immune function or mild-to-moderate immunosuppression may selectively receive probiotics or FMT following standard therapy, accompanied by periodic microbiota monitoring. In contrast, patients with severe immunosuppression (e.g., neutropenia or bone marrow suppression during chemotherapy or radiotherapy) require cautious evaluation of the benefit–risk balance before intervention.

Advanced computational approaches may further accelerate this field. For instance, graph neural network models can be used to analyze microbial co-occurrence networks and efficiently identify pathogenic and protective taxa (79). Such approaches may guide strain selection and microbial formulation design. Ultimately, integrating genomic, serological, and microbiome features into multi-omics artificial intelligence models could enable simultaneous prediction of therapeutic response and adverse event risk (80, 81). This strategy may provide personalized decision support for patients with different immune states, maximizing the benefits of microbiota modulation while minimizing safety risks. Through these efforts, the gut microbiota may transition from a previously overlooked “bystander” to an active participant in precision prevention and treatment of cervical cancer.

The central panel (Mechanisms & Challenges) illustrates both pathological mechanisms and practical barriers. Gut microbiota dysbiosis leads to reduced SCFA production and NK cell exhaustion. The resulting collapse of systemic immunity, combined with disruption of the vaginal microbial barrier, converts transient HPV infection into persistent infection and drives disease progression from cervical intraepithelial neoplasia (CIN) to invasive cervical cancer. This panel also highlights four major challenges in the field: the absence of longitudinal cohorts integrating gut, vaginal, and immune datasets; the difficulty of controlling confounding factors such as sexual behavior, hormonal status, diet, and ethnicity; the lack of standardized protocols for fecal microbiota transplantation (FMT) and probiotic interventions; and the uncertain transfer efficiency between systemic and intralesional immunity.

The right panel (The Call for Management) proposes a paradigm shift, repositioning the gut microbiota from a previously overlooked “bystander” to an active “participant” in precision prevention and management of cervical cancer. A vertical stepwise timeline outlines a four-stage clinical management framework: (1) during screening, microbiota-derived indicators serve as auxiliary biomarkers for risk stratification; (2) at the precancerous (CIN) stage, probiotics or microbiota-based vaccines act as synergistic targets to enhance HPV clearance; (3) in advanced disease, gut microbiota modulation may function as a sensitizing strategy for immune checkpoint inhibitors such as PD-1 blockade; and (4) following chemoradiotherapy, microbiome restoration may represent a key intervention window to improve long-term outcomes.

## Data Availability

The original contributions presented in the study are included in the article/supplementary material. Further inquiries can be directed to the corresponding author.
